# Epoxy Composites Reinforced with Sol–Gel Synthesized Alumina–Silica, Alumina, and Natural Silica Fillers: Comparative Mechanical Performance

**DOI:** 10.3390/gels12050408

**Published:** 2026-05-08

**Authors:** Milica Marković, Marija M. Vuksanović, Miloš Petrović, Željko Radovanović, Radmila Jančić Heinemann, Vera Obradović

**Affiliations:** 1 Faculty of Technology and Metallurgy, University of Belgrade, 11000 Belgrade, Serbia; mpetrovic@tmf.bg.ac.rs; 2 Department of Chemical Dynamics and Permanent Education, VINČA Institute of Nuclear Sciences—National Institute of the Republic of Serbia, University of Belgrade, 11351 Belgrade, Serbia; marija.vuksanovic@vin.bg.ac.rs; 3 Innovation Center of Faculty of Technology and Metallurgy in Belgrade, 4 Karnegijeva Street, 11120 Belgrade, Serbia; zradovanovic@tmf.bg.ac.rs (Ž.R.); vobradovic@tmf.bg.ac.rs (V.O.)

**Keywords:** epoxy composites, sol–gel alumina, rice husk silica, diatomaceous earth silica, hybrid alumina–silica fillers, mechanical properties, flexural strength, tensile strength

## Abstract

Epoxy resins are widely used thermosetting polymers, but their limited toughness and flexural resilience restrict broader applications. In this study, diglycidyl ether of bisphenol A (DGEBA) epoxy was reinforced with 5 wt.% ceramic fillers of different origins: sol–gel alumina calcined at 550 °C (γ-Al_2_O_3_) and 1000 °C (α-Al_2_O_3_), silica derived from rice husk, silica from diatomaceous earth, and a hybrid alumina–silica mixture prepared by sol–gel and calcined at 1000 °C. Fillers were structurally characterized by X-ray diffraction (XRD), Fourier transform infrared spectroscopy (FTIR), and field-emission scanning electron microscopy (FESEM). Mechanical properties were evaluated through tensile (ASTM D638) and flexural (ASTM D790) testing. All reinforcements enhanced the performance of neat epoxy. γ-Al_2_O_3_ provided superior tensile reinforcement compared to α-Al_2_O_3_, underscoring the importance of particle morphology and surface reactivity. The hybrid alumina–silica filler achieved the highest flexural strength of 50.6 MPa, compared to 9.91 MPa for the neat epoxy. Bio-derived silica showed improved flexural properties, although its tensile reinforcement was less pronounced compared to the sol–gel derived fillers. These results establish clear structure–property relationships and confirm that filler phase, morphology, and calcination temperature critically govern the mechanical performance of epoxy composites.

## 1. Introduction

Due to their superior dimensional stability, chemical resistance, and excellent adhesion, epoxy resins have become indispensable in high-performance sectors such as aerospace, automotive engineering, and advanced electronics. However, their inherent brittleness, stemming from a high cross-link density, remains a critical challenge that limits their energy absorption capacity and impact resistance. To address these deficiencies, various ‘toughening’ strategies for agricultural waste, particularly rice husk, have been extensively studied as sustainable precursors for bio-silica, focusing on the incorporation of rigid ceramic fillers to promote energy-dissipating mechanisms such as crack deflection, micro-voiding, and particle bridging [1]. Diglycidyl ether of bisphenol A (DGEBA), which is made by condensation of bisphenol A with epichlorohydrin, is the most widely used commercial epoxy. Even though pure epoxy resins are useful and adaptable, their limited flexural strength and hardness restrict broader technical applications [1].

Ceramic fillers, such as silica (SiO_2_) and alumina (Al_2_O_3_), have been added to epoxy matrices to overcome these restrictions. Prior research has shown that silica and alumina nanoparticles can greatly improve the mechanical, thermal, and dielectric properties of epoxy composites despite the fact that particle agglomeration and poor dispersion may lower their impact strength [2,3]. The sol–gel-produced alumina nanofillers exhibit conceptually encouraging reinforcing effects because of their controlled morphology and surface chemistry [4]. Sol–gel synthesis offers a distinct advantage over conventional methods by allowing molecular-level control over filler purity, homogeneity, and textural properties [5]. For alumina reinforcements, the calcination temperature plays a pivotal role in determining the final crystalline phase, ranging from high-surface-area transitional phases like γ-Al_2_O_3_, η-Al_2_O_3_, κ-Al_2_O_3_ to the chemically stable and mechanically rigid α-Al_2_O_3_. Those effects are observed in acrylic polymer matrix composites [6,7]. Understanding how these phase transformations influence the filler-matrix interface is essential for optimizing the mechanical load transfer in epoxy systems. Nano-alumina has been proven to influence the fracture toughness of epoxy composites even at low filler content [8].

Similarly, it has been reported that silica derived from sustainable sources, like rice husk, enhances the mechanical properties of acrylic composites [9] and fracture toughness of epoxy resins [10,11]. Due to its distinctive porous morphologies that promote interfacial bonding, diatomaceous earth, a naturally occurring biogenic material with a high silica concentration, has also been investigated as a reinforcement [12]. In recent years, the shift toward sustainable materials has intensified interest in bio-derived silica sources. Rice husk, a prevalent agricultural byproduct, can be processed into high-purity amorphous or crystalline silica through controlled acid leaching and calcination, offering a low-cost and eco-friendly alternative to synthetic fumed silica. Similarly, diatomaceous earth provides a unique biogenic silica structure characterized by intrinsic porosity and complex frustule morphologies, which can enhance mechanical interlocking at the composite interface [12]. 

This study systematically compares sol–gel-synthesized γ-Al_2_O_3_ and α-Al_2_O_3_, bio-derived silica from rice husk, diatomaceous earth silica, and a hybrid alumina–silica filler AluSil within the same epoxy matrix under identical processing conditions, thereby establishing direct structure–property correlations. Alumina and silica hybrid fillers have gained significant interest due to their ability to simultaneously improve stiffness and toughness while maintaining high thermal stability. According to studies [13], these systems can produce better mechanical properties than single-component fillers if dispersion is appropriately controlled.

By combining the high hardness of alumina with the crack-deflecting capabilities of silica, hybrid systems like alumina–silica (AluSil) can induce synergistic effects that surpass the performance of single-filler composites. The efficacy of such systems, however, is heavily dependent on the filler ratio and the development of stable crystalline phases produced during thermal treatment [14], as it has been shown for acrylic resin.

The aim of this work is to quantitatively assess how filler type and calcination temperature affect the flexural and tensile properties of DGEBA epoxy composites, and to demonstrate the potential of bio-derived and hybrid ceramic reinforcements for sustainable composite design. Unlike previous studies that examined individual fillers, our work provides a direct comparison under identical conditions, confirming the superior tensile reinforcement of γ-Al_2_O_3_ over α-Al_2_O_3_, and the synergistic toughening effect of hybrid alumina–silica fillers.

## 2. Results and Discussion

### 2.1. Structural Analysis of Synthesized Particles

**Figure 1:**
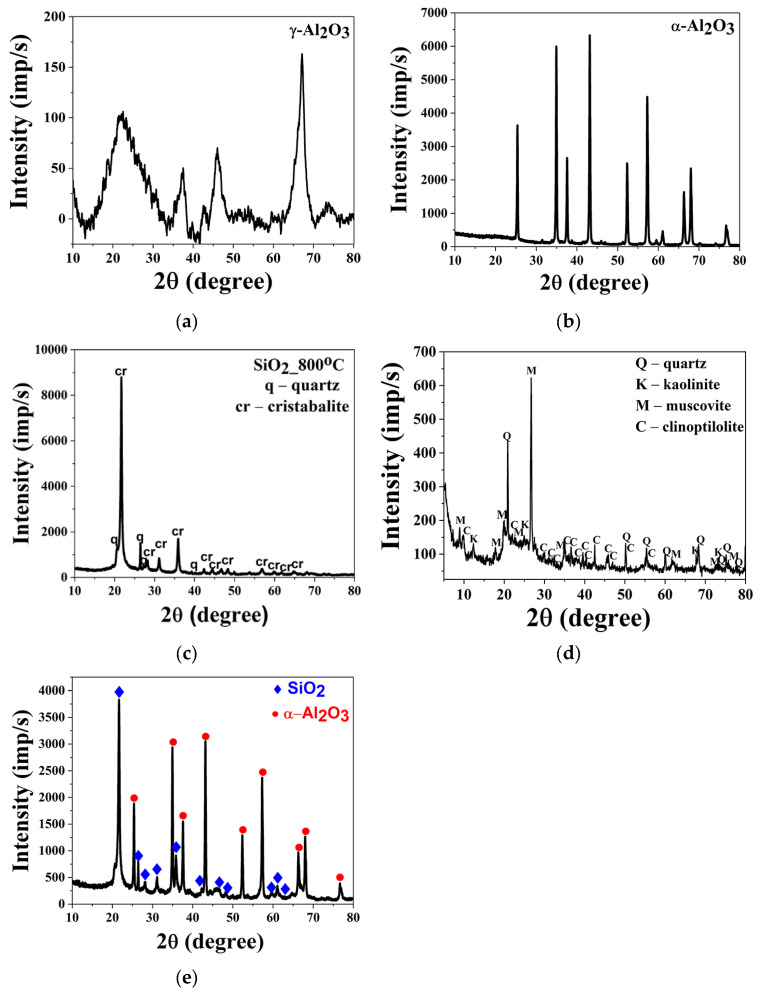
XRD diffractograms of particles: (**a**) γ-Al_2_O_3_, (**b**) α-Al_2_O_3_, (**c**) SiO_2_ from rice husk, (**d**) SiO_2_ from diatomaceous earth, and (**e**) mixed Al_2_O_3_ and SiO_2_.

The structural analysis of reinforcement particles was carried out using X-ray diffraction (XRD), as presented in Figure 1.

Alumina particles calcined at 550 °C (Figure 1a) exhibited broad, low-intensity reflections in the 2θ range of 15–35°. The XRD pattern exhibits all the characteristic reflections of γ-Al_2_O_3_, including the broad peaks at 2θ = 25.3° (311), 37.8° (400), 45.8° (440), and 66.9° (622), which correspond to the standard PDF cards (10-0425, 29-0063, and 01-074-2206). The large peak widths and reduced intensities indicate low crystallinity, which is consistent with the transitional alumina phase obtained after calcination at 550 °C. This result is fully logical, as γ-alumina at this temperature remains poorly ordered compared to the highly crystalline α-Al_2_O_3_ (PDF 46-1212 and 01-076-7777). The XRD pattern of the sample shows the characteristic reflections of α-Al_2_O_3_ (corundum), with the strongest peaks observed at 2θ = 35.1° (104) and 57.5° (116), along with additional reflections at 25.6° (012), 37.9° (110), 43.4° (113), 52.6° (024), 61.3° (018), 66.5° (214), 68.2° (300), 77.1° (10 0, 119), and 87.6° (2 10). These match well with the standard PDF cards for corundum, including PDF 46-1212 and PDF 01-076-7777, confirming the presence of α-alumina.

The XRD pattern of silica obtained from rice husk and calcined at 800 °C (Figure 1c) revealed the presence of two crystalline phases: dominant cristobalite, with its most intense peak at 2θ ≈ 22°, and minor quartz. The XRD pattern reveals the presence of both cristobalite and quartz phases. Cristobalite is identified by its strong reflection at 2θ = 22.0° (101), along with weaker peaks at 36.0° (200), 42.6° (211), and 49.6° (220), in agreement with PDF cards 39-1425 and 82-0512. Quartz is confirmed by the sharp reflection at 26.6° (101), together with peaks at 20.9° (100), 36.5° (110), 39.5° (102), and 50.1° (112), matching PDF cards 46-1045 and 85-0796. The relatively broad and low-intensity peaks indicate poor crystallinity, which is consistent with the calcination conditions and the coexistence of these silica polymorphs. In contrast, silica derived from diatomaceous earth (Figure 1d) exhibited a broad diffraction band in the 2θ region of 15–28°, characteristic of amorphous SiO_2_. The most intense peak at ≈26.7° corresponded to muscovite, while quartz reflections were observed at ≈ 20.9° and 26–27°. Additional phases such as kaolinite and clinoptilolite were also detected, indicating the presence of clay and zeolite impurities [15,16,17].

The diffractogram of AluSil particles synthesized in a 3:2 Al_2_O_3_–SiO_2_ ratio and calcined at 1000 °C (Figure 1e) revealed diffraction maxima corresponding to α-Al_2_O_3_ and cristobalite SiO_2_. Typical mullite reflections expected at 2θ ≈ 16.5°, 25.8°, 32.6°, and 41.7° appeared only as weak signals, suggesting that mullite crystallization was at an initial stage. The absence of significant mullite formation is attributed to the relatively low calcination temperature and the introduction of SiO_2_ as pre-formed particles, which restricted the extent of reaction [14,18]. The α-Al_2_O_3_ reflections exhibited high intensities, confirming its dominant presence, while a broad band in the 20–25° region indicated residual glassy or poorly crystalline silicate phases.

### 2.2. Morphological Analysis of Reinforcements

**Figure 2:**
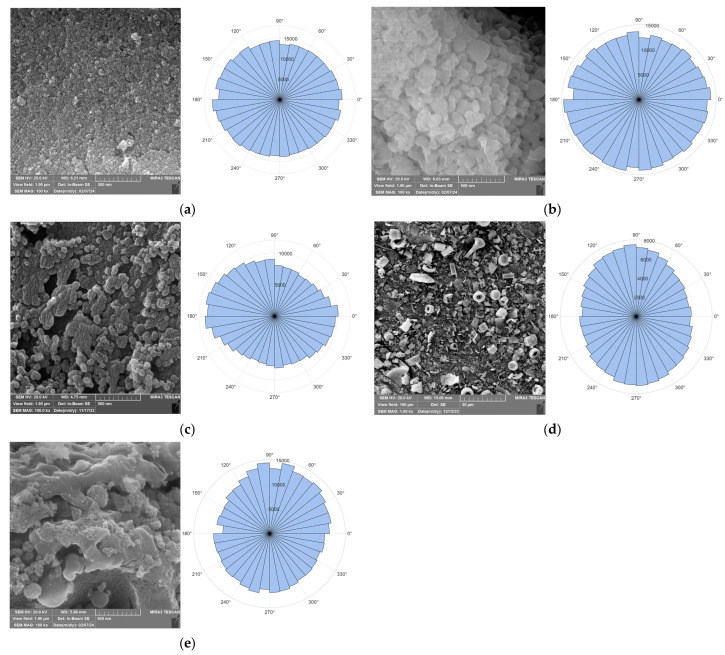
SEM images and radar Rose diagrams of the reinforcing particles: (**a**) sol–gel alumina calcined at 550 °C, (**b**) sol–gel alumina calcined at 1000 °C, (**c**) rice-husk-derived silica calcined at 800 °C; (**d**) diatomaceous earth from Kolubara basin, and (**e**) synthesized mixed particles of silica and alumina.

Figure 2 presents the SEM images of particles used as reinforcements. The SEM micrograph of alumina particles made from aluminum chlorohydrate (ACH) using the sol–gel method and calcined at 550 °C (Figure 2a) showed the creation of a finely dispersed structure with distinct nanograin shape. The micrograph revealed a very uniform surface made up of nanometric primary particles forming porous, loose agglomerates without distinct grain boundaries. According to Attil [19], such morphology is typical for alumina obtained by the sol–gel route after calcination at moderate temperatures, where the material typically remains in an amorphous or weakly crystalline γ-state, marked by limited diffusion processes and minimal grain growth. The calcination of particles at 1000 °C, a temperature frequently used to produce α-Al_2_O_3_, showed visible morphological changes, increased crystallinity, grain expansion, and densification. 

The SEM image of rice husk-derived silica particles, calcined at 800 °C (Figure 2c), shows distinctive aggregation. Here, the particle sizes varied from a few micrometers to several hundred nanometers. 

The morphological diversity of diatomaceous earth reflects variability in frustule architecture (siliceous cell walls of diatoms), which is dictated by species, depositional environment, and subsequent processing. As illustrated in Figure 2d, the powder exhibits pronounced porosity and exceptionally low bulk density, with preserved and fragmented plate-like and cylindrical structures containing circular and hexagonal pores. The functional performance of diatomaceous earth is intrinsically linked to this geometric complexity and local morphological heterogeneity. Notably, in composite fabrication studies, the diversity of structural motifs exerts a direct influence on specific surface area and adsorption behavior [20]. 

Strong agglomeration and the development of a complex, heterogeneous microstructure are visible in the SEM picture of the AluSil particle mixture (Figure 2e). The image analysis revealed the existence of tiny primary particles loosely joined, as well as irregularly shaped aggregates with comparatively rough surfaces. The overall microstructure of the AluSil combination was greatly impacted by the addition of SiO_2_ derived from rice husks. Following thermal treatment at 1000 °C, bio SiO_2_ crystallized into the β cristobalite phase, which changed into metastable α cristobalite when cooled below 270 °C [14,21]. This interpretation aligns with the XRD analysis of the AluSil particles. 

SEM images were subjected to image analysis to evaluate individual particle morphology [22]. To quantify microstructural features, an orientation analysis based on pixel intensity gradients was performed. The original SEM micrographs reveal a clear morphological evolution: from fine-grained nanoparticles (alumina at 550 °C and bio-silica) to coarser aggregates in the AluSil and alumina calcined at 1000 °C samples. To evaluate the filler distribution capacity, orientation analysis was performed using pixel intensity gradients. The resulting Rose diagram (Figure 2) indicates a lack of preferential orientation within the analyzed cross-sections. While this method is based on 2D image processing rather than a 3D structural standard, the observed distribution suggests a high degree of structural uniformity, which opens the possibility of incorporation in the composite as a homogeneous structural part.

### 2.3. FTIR Analysis of Composite Samples

**Figure 3:**
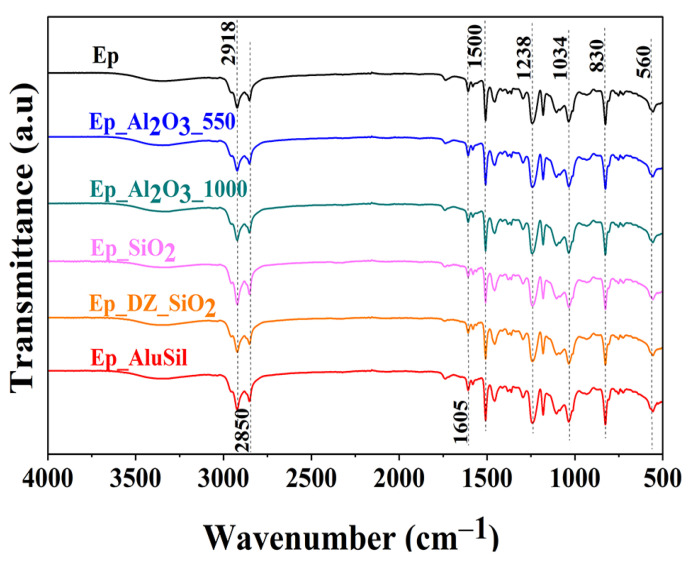
FTIR spectra of composite materials.

The FTIR spectra of the neat epoxy and its composites are shown in Figure 3. The neat resin exhibits characteristic bands of a bisphenol A-based system. A broad band at 3600–3200 cm^−1^ corresponds to O–H stretching, originating from both the curing process and the potential hydration of the hydrophilic ceramic fillers. The peak at ~3055 cm^−1^ is assigned to aromatic C–H stretching, while bands at 2918–2850 cm^−1^ and 1605–1500 cm^−1^ relate to aliphatic/aromatic C–H and benzene ring vibrations, respectively. Ether linkages (C–O–C and C–O) appear in the 1238–1034 cm^−1^ range, and the low intensity of the epoxy ring band at 921 cm^−1^ confirms successful curing. Out-of-plane C–H vibrations of substituted aromatic rings are observed at 830–803 cm^−1^ and 752–560 cm^−1^ [23]. In the composite spectra, the matrix bands remain dominant, suggesting that the 5 wt. % filler loading did not significantly alter the chemical structure. Specific filler-matrix interactions, such as Si–O–C and Al–O–C, were not clearly resolved due to overlapping with the epoxy signals at this concentration.

### 2.4. Mechanical Testing of Composites

#### 2.4.1. Flexural Testing

Flexural strength testing revealed significant differences in results between the neat epoxy and the composites reinforced with various fillers (Table 1). The neat epoxy resin exhibited a flexural strength of 9.91 MPa, serving as the baseline. Incorporation of fillers generally improved mechanical performance, though the extent of reinforcement depended strongly on filler type and processing conditions. 

The Ep_AluSil composite achieved the highest flexural strength of 50.6 MPa, corresponding to a 410% increase compared to neat epoxy. This enhancement can be attributed to the synergistic effect of alumina and silica phases, which provide both rigidity and improved interfacial bonding. Similar improvements in hybrid fillers have been reported in the literature [14].

Composites reinforced with bio SiO_2_ (42.58 MPa) and DZ-SiO_2_ (33.43 MPa) also exhibit notable increases in flexural strength. The improvement is linked to the porous morphology and surface chemistry of silica particles, which enhance adhesion with the epoxy matrix. 

Alumina fillers unveiled contrasting behavior depending on calcination temperature. Ep_Al_2_O_3__550 (33.87 MPa) showed a clear improvement over neat epoxy, while Ep_Al_2_O_3__1000 (21.96 MPa) was only marginally higher than the baseline flexural strength. The reduced reinforcement efficiency at higher calcination temperatures is explained by grain coarsening and a decrease in the surface reactivity of α-Al_2_O_3_, which limits interfacial bonding [24]. 

Overall, the results confirmed that filler type, morphology, and processing conditions critically influence flexural performance. The hybrid AluSil filler provided the most pronounced improvement, while bio-derived silica offered a sustainable alternative with strong reinforcement potential. Alumina calcined at lower temperatures proved more effective than its high-temperature counterpart, highlighting the importance of fine particle size and reactive surfaces for optimal composite performance.

The statistical analysis of flexural data (Table 2) confirmed that all reinforced composites exhibited significantly higher modulus and strength compared to neat epoxy. The ANOVA analysis performed in MATLAB software (under the Campus-Wide (CW) license (MathWorks, Natick, MA, USA) version R2024b) confirmed that all reinforcements improve the mechanical behavior of composites in flexion. The Ep_AluSil composite achieved the highest flexural modulus (1688.09 ± 278.34 MPa) and strength (50.60 ± 3.71 MPa), representing ~6× and ~5× improvements, respectively, to the matrix material. However, its relatively large standard deviation indicates variability in particle dispersion. Ep_SiO_2_ showed the second-highest strength (42.58 ± 0.68 MPa) with the lowest variability, suggesting reinforcement with predictable reinforcing action. In contrast, Ep_Al_2_O_3__1000 exhibited the weakest performance among composites (966.73 ± 152.47 MPa modulus; 21.96 ± 3.16 MPa strength), lower than Ep_Al_2_O_3__550, confirming that higher calcination temperatures reduce reinforcement efficiency.

Overall, comparisons demonstrate that AluSil provides the strongest reinforcement in terms of modulus and strength, while bio SiO_2_ offers the most consistent and balanced improvement with preserved ductility. The negative effect of high-temperature calcination on alumina was statistically significant, underscoring the importance of filler morphology and surface chemistry in determining composite performance.

#### 2.4.2. Tensile Test Results

Table 2 shows the results of tensile testing of all specimens. As expected, the tensile strength and the modulus values were generally lower than those obtained in flexural testing. This difference arises from the loading mode: in tensile testing, the specimen is uniformly stressed in tension, whereas in flexural testing, the specimen experiences a combination of tensile, compressive, and shear stresses under three-point bending. The flexural modulus obtained from flexural testing represents a combined structural response, whereas tensile testing isolates the intrinsic tensile modulus of the material [25].

**Table 2:** The tensile test results.

Specimen	Tensile Strength (MPa)	Tensile Modulus (MPa)	Deformation %
Ep	15.81 ± 0.65	494.38 ± 30.69	17.4
Ep_AluSil	36.77 ± 0.24	820.28 ± 28.40	7.74
Ep_Al_2_O_3__550	37.28 ± 0.34	854.32 ± 20.35	8.68
Ep_Al_2_O_3__1000	22.00 ± 2.20	615.22 ± 54.05	8.57
Ep_SiO_2_	23.93 ± 3.48	648.82 ± 15.41	6.12
Ep_DZ_SiO_2_	23.34 ± 3.04	561.30 ± 34.44	7.99

Sample geometry influences tensile testing less than bending. Reinforcements transfer loads more effectively under flexural conditions, whereas in tensile loading, agglomeration or weak interfacial adhesion can have more pronounced negative effects. In bending, internal imperfections may be less critical due to distributed stresses, whereas in tensile testing, even minor defects in specimen preparation can initiate fracture and reduce both modulus and measured strength [26]. Overall, the results confirmed that the tensile properties of epoxy composites are more sensitive to microstructural imperfections and filler dispersion than the flexural properties. This highlights the importance of achieving uniform particle distribution and strong interfacial bonding to maximize tensile performance.

Compared to neat epoxy, the best improvement in tensile modulus was achieved with Al_2_O_3__550 particles (≈1.73×) and in tensile strength with Al_2_O_3__550 (≈2.36×). Unlike the flexural properties, where Ep_AluSil showed the highest values, the tensile performance followed a different trend, likely due to different stress-transfer mechanisms under tension. 

Raising the calcination temperature of alumina (γ → α transition) led to a 29% decrease in tensile modulus, and 41% in strength. This decline is attributed to structural and surface transformations: γ-Al_2_O_3_ retains a porous, highly dispersed morphology with large surface area, enabling efficient stress transfer, while α-Al_2_O_3_ undergoes sintering and loses reactive OH groups, resulting in poor adhesion [27]. Consequently, Ep_Al_2_O_3__1000 showed the largest standard deviation in tensile modulus, indicating high heterogeneity. 

Ep_AluSil composites exhibited tensile properties close to Ep_Al_2_O_3__550, with slightly lower modulus and strength but reduced strain, indicating a different reinforcement mechanism for the sol–gel-synthesized hybrid AluSil and natural silica composites compared to Ep_Al_2_O_3__1000. However, AluSil showed significant improvement. The presence of SiO_2_ functioned as a physical separator, preventing alumina agglomeration and promoting more homogeneous dispersion, while also enhancing compatibility with the epoxy matrix.

By contrast, composites with bio-SiO_2_ and DZ-SiO_2_ exhibited the lowest tensile modulus and strength, comparable to Ep_Al_2_O_3__1000. However, they retained greater strain capacity compared to Ep_Al_2_O_3__550 composites.

The tensile test results (Table 2) revealed significant differences between neat epoxy and reinforced composites. Neat epoxy exhibited the lowest tensile modulus (494.38 ± 30.69 MPa) and strength (15.81 ± 0.65 MPa). The Ep_Al_2_O_3__550 composite achieved the highest tensile modulus (854.32 ± 20.35 MPa) and strength (37.28 ± 0.34 MPa), representing ~1.73× and ~2.36× improvements over the neat epoxy. Ep_AluSil showed similar values (820.28 ± 28.40 MPa modulus; 36.77 ± 0.24 MPa strength), comparable to Ep_Al_2_O_3__550.

By contrast, Ep_Al_2_O_3__1000 exhibited reduced performance (615.22 ± 54.05 MPa modulus; 22.00 ± 2.20 MPa strength), significantly lower than Ep_Al_2_O_3__550, confirming that elevated temperature calcination decreases reinforcement efficiency. Silica fillers provided moderate improvements: Ep_SiO_2_ (648.82 ± 15.41 MPa; 23.93 ± 3.48 MPa) and Ep_DZ_SiO_2_ (561.30 ± 34.44 MPa; 23.34 ± 3.04 MPa) were similar.

Overall, comparisons demonstrate that γ-Al_2_O_3_ (550 °C) is the most effective reinforcement for tensile properties, while AluSil provides a balanced performance close to γ-Al_2_O_3_. Silica fillers contribute less to modulus and strength but preserve ductility. The negative effect of α-Al_2_O_3_ formation at 1000 °C was clearly evident, underscoring the importance of the filler phase and surface chemistry in determining tensile reinforcement efficiency.

#### 2.4.3. Proposed Reinforcement Mechanism of Alumina–Silica Hybrid Composites 

**Figure 4:**
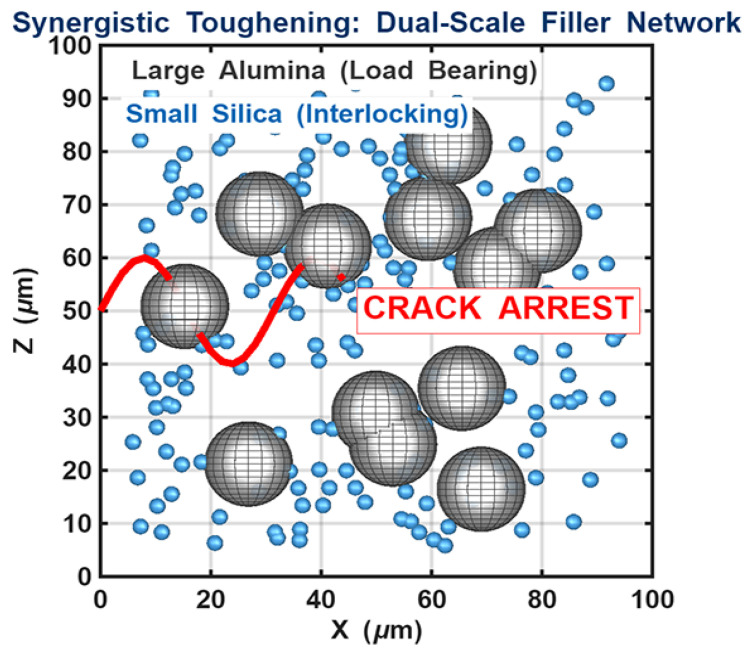
Conceptual schematic representation of the dual-scale reinforcement mechanism, showing the distribution of micro- and nano-sized fillers within the epoxy matrix (generated via MATLAB routine).

The experimental data indicate a clear enhancement in mechanical stability for the hybrid AluSil and natural silica-reinforced systems. As conceptually illustrated in Figure 4, the synergistic interaction between the alumina and silica phases is the primary mechanism behind the optimized flexural properties. Within this dual-scale network, large alumina particles function as a load-bearing skeleton, while smaller silica particles are distributed within the inter-particle voids. This hierarchical architecture creates a “shielding effect” that reduces inter-particle distance and prevents excessive local deformation of the epoxy matrix, ensuring a more uniform stress distribution and preventing premature micro-crack initiation.

**Figure 5:**
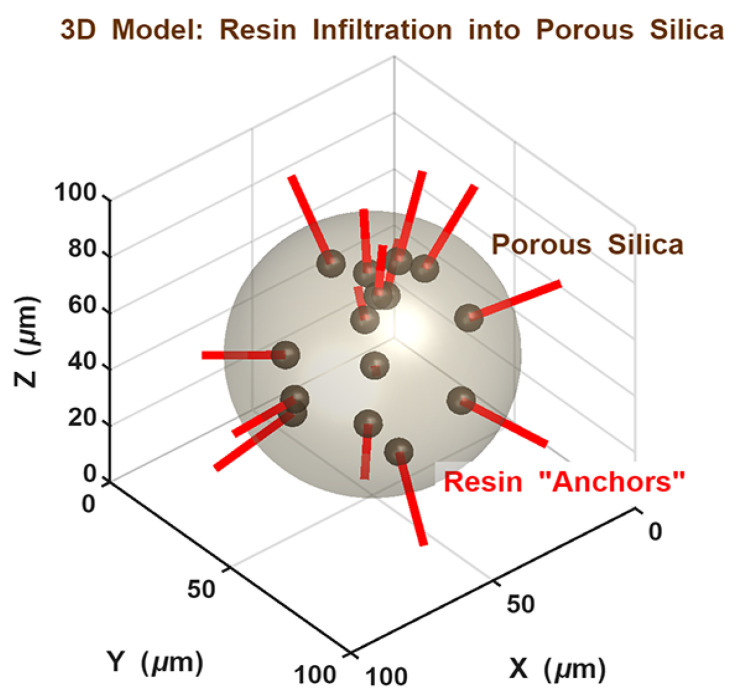
Conceptual schematic illustration of the interfacial bonding mechanism: the red features represent the cured epoxy resin infiltrating the internal pore network (dark spheres) of the bio silica, creating a mechanical interlocking effect.

This structural integrity is further enhanced by mechanical interlocking, as schematically shown in Figure 5. The high flexural resilience, particularly in 5 wt.% bio-silica and porous γ-Al_2_O_3_ samples, is attributed to the infiltration of the cured resin into the internal pore network of the particles. The formation of these “resin anchors” prevents particle pull-out and facilitates superior load transfer compared to non-porous systems, effectively ensuring the integrity of the hybrid filler network under mechanical stress.

**Figure 6:**
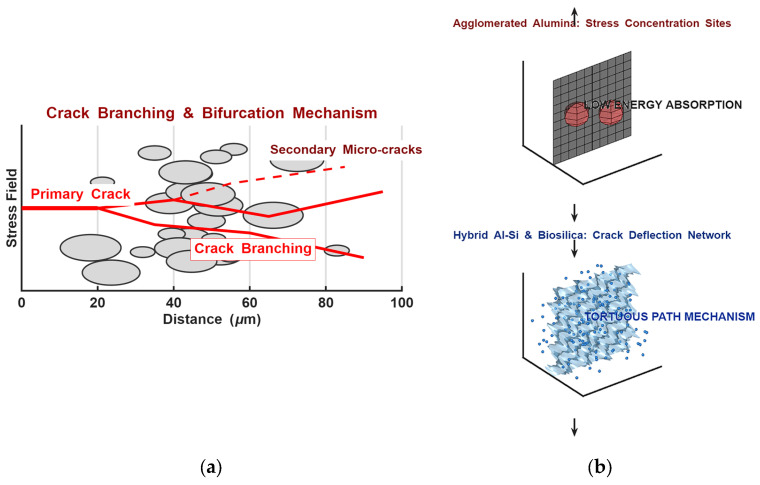
(**a**) Conceptual schematic representation of crack propagation behavior; (**b**) comparison of failure modes: linear crack path in single-filler agglomerated systems (upper panel) vs. crack branching and bifurcation mechanisms in hybrid AluSil epoxy composites (lower panel).

While Figure 6a,b conceptually illustrate how filler networks might influence crack paths, the present data (flexural and impact testing) do not allow direct conclusions about fracture-toughness mechanisms, such as crack deflection or branching. These schematic representations are included only as possible interpretations.

As shown in the upper panel of Figure 6b, composites reinforced with single-phase alumina particles (α-Al_2_O_3_ and γ-Al_2_O_3_) tend to form macro-agglomerates. These clusters, arising from high surface energy and limited interfacial wetting, act as stress concentration sites. Under loading, failure in these systems often follows relatively direct paths through the matrix and around poorly bonded agglomerates, which is consistent with the lower flexural resilience and more brittle character observed experimentally.

In contrast, the lower panel of Figure 6b illustrates the improved dispersion achieved in the sol–gel synthesized hybrid AluSil and natural silica composites. The more uniform distribution of fillers supports a denser particle network and stronger interfacial bonding. This microstructural arrangement is associated with higher flexural resilience in these systems. While schematic representations suggest that such networks could influence crack paths, the present data (flexural and impact testing) do not allow for direct conclusions about fracture-toughness mechanisms. The figures are therefore included as conceptual illustrations rather than evidence of specific crack deflection or energy dissipation processes.

#### 2.4.4. Fracture Surface Analysis

**Figure 7:**
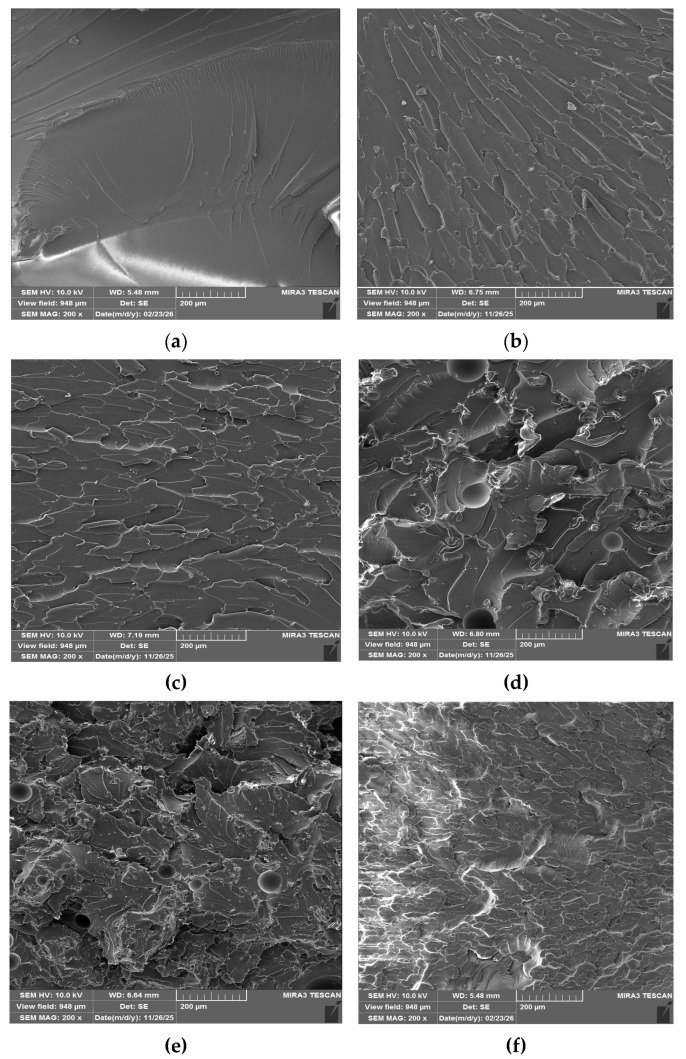
Fracture surfaces of specimens (**a**) neat epoxy, (**b**) sol–gel alumina calcined at 550 °C, (**c**) sol–gel alumina calcined at 1000 °C, (**d**) rice-husk-derived silica calcined at 800 °C, (**e**) diatomaceous earth from Kolubara basin, and (**f**) synthesized mixed particles of silica and alumina.

The fracture surfaces of all the specimens after their complete fracture into two parts in the tensile testing are depicted in Figure 7.

The fracture analysis is presented by the proper SEM images and can be described for each of them in the following way:

- Figure 7a (Neat epoxy): Smooth fracture surface, typical brittle failure with limited crack deflection.
- Figure 7b (Ep_Al_2_O_3__550): long parallel striations show limited flexibility and brittle fracture. Fracture shows greater roughness and opposition to crack growth, though γ-Al_2_O_3_ improved adhesion, because of OH groups and moderate roughness, indicating partial crack deflection.
- Figure 7c (Ep_Al_2_O_3__1000): layered fracture surface featuring zones that are both ductile and brittle: decreased roughness relative to the sample at 550 °C, indicating increased crystallinity and decreased adhesion. As stress concentrators. Agglomeration causes early crack initiation.
- Figure 7d (Ep_SiO_2_): rough, uneven surface with granular and fibrous textures. Increased toughness and crack deflection are encouraged by good particle dispersion. In line with better mechanical qualities, Si–OH groups promote adhesion.
- Figure 7e (Ep_DZ_SiO_2_): rough fracture with voids and porous areas. Dispersion was lower than in bio-SiO_2_, resulting in minimal crack deflection and brittle fracture. Reduced surface area and mineralized biogenic structure diminish the effectiveness of reinforcement.
- Figure 7f (Ep_AluSil): uneven aggregates with a complex, diverse morphology. Phases Al_2_O_3_ and SiO_2_ cannot be separated; however, partial sintering is visible. Strength is increased by the creation of cristobalite, while strain is still low.

Fracture surfaces show distinct variations in the effectiveness of reinforcing. Compared to α-Al_2_O_3_ (1000 °C), which becomes brittle due to sintering and reduced OH groups. γ-Al_2_O_3_ (550 °C) exhibits superior adhesion and toughness. AluSil benefits from SiO_2_ dispersion by striking a balance between toughness and strength. Among silica fillers, DZ-SiO_2_ is less effective because of its porosity and poor dispersion, whereas Bio-SiO_2_ offers the best roughening of the fracture surface. In general, silica fillers increase strain. Alumina fillers increase tensile strength, and AluSil provides a compromise suitable for mixed loading conditions.

**Figure 8:**
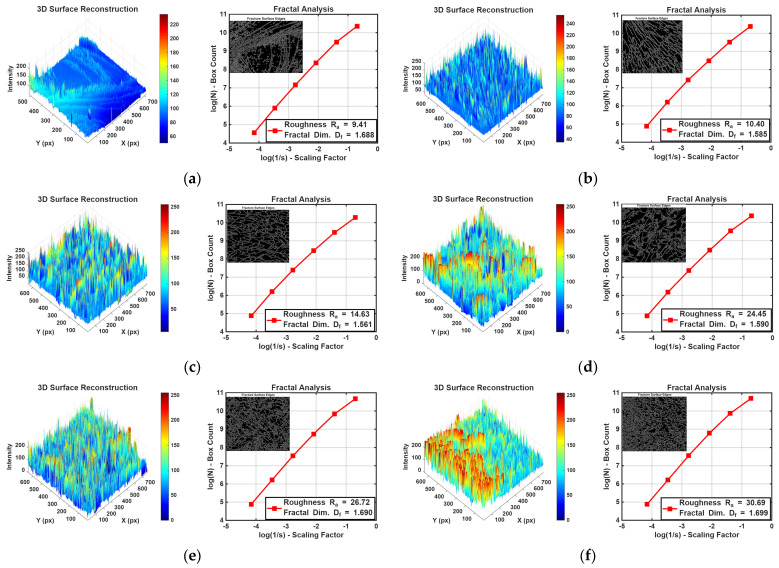
Quantitative fractographic analysis of the fracture surface for: (**a**) neat epoxy, (**b**) sol–gel alumina calcined at 550 °C, (**c**) sol–gel alumina calcined at 1000 °C, (**d**) rice-husk-derived silica calcined at 800 °C, (**e**) diatomaceous earth from Kolubara basin, and (**f**) synthesized mixed particles of silica and alumina.

The evolution of the fracture surface morphology was quantitatively assessed through 3D intensity-based reconstruction and fractal analysis, providing a numerical correlation between filler incorporation and matrix topography, as illustrated in Figure 8.

The 3D topographical maps (left panels) offer a clear visualization of the fracture landscape transformation. The neat epoxy matrix (Figure 7a) exhibits a relatively smooth and quasi-planar surface, which is reflected in its low arithmetical mean roughness (R_a_ = 9.41 a.u.). In contrast, the addition of reinforcement particles induces a progressive transition toward rugged terrain, characterized by prominent peaks and valleys. This is most evident in the hybrid composite (Figure 8f), where the 3D reconstruction reveals a highly irregular and chaotic morphology reaching a maximum roughness of R_a_ = 30.69. The geometric complexity and space-filling capacity of these surfaces were further quantified using the fractal dimension (D_f_) derived from the box-counting plots (right panels). 

It should be emphasized that the R_a_ values reported herein represent image-based intensity metrics (optical roughness) derived from gray-scale variations, rather than topographic surface roughness measured by conventional profilometry. These values, along with the fractal dimension (D_f_), are used as comparative indicators of fracture surface complexity.

All reinforced samples maintained high D_f_ values. ranging from 1.561 to 1.699 and indicating a significant deviation from Euclidean geometry. The simultaneous occurrence of the highest roughness and the highest fractal dimension (D_f_ = 1.699) in Figure 8f suggests the potential for a crack deflection mechanism. Deviation from a linear path significantly increases the total fracture surface area and enhances energy dissipation, which enables improved mechanical performance observed in the hybrid composite system.

The observed increase in both *R_a_* and *D_f_* values, supported by 3D surface reconstructions and log–log fractal plots, suggests that the incorporation of the hybrid filler system may facilitate crack deflection and elevate the energy required for fracture. These findings indicate a potential enhancement in the mechanical robustness of the composite, attributable to the complex interplay of morphological features introduced by the filler.

**Figure 9:**
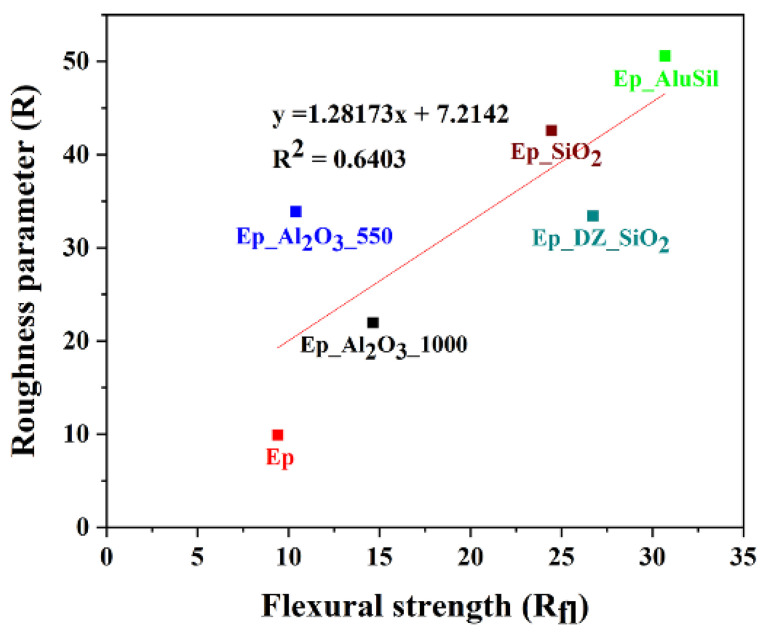
Linear regression analysis of roughness parameter (R) vs. flexural strength (R_fl_) for epoxy-based composites.

The correlation of the fracture roughness to the bulk property measurement was done with the attempt to correlate the microstructural feature to the measurable behavior of the composite. The correlation was possible for roughness and flexural strength. The correlation coefficient obtained was R^2^ = 0,63. If the correlation is done only for reinforcements with very fine particles, excluding diatomaceous earth, which has micrometer-sized particles, the correlation is R^2^ = 0.74, suggesting that this filler behaves slightly differently (Figure 9). Other measured properties showed poor correlation, suggesting that the flexural strength is the property that enables the composite to exhibit the most informative property.

#### 2.4.5. Controlled Energy Impact Testing

**Figure 10:**
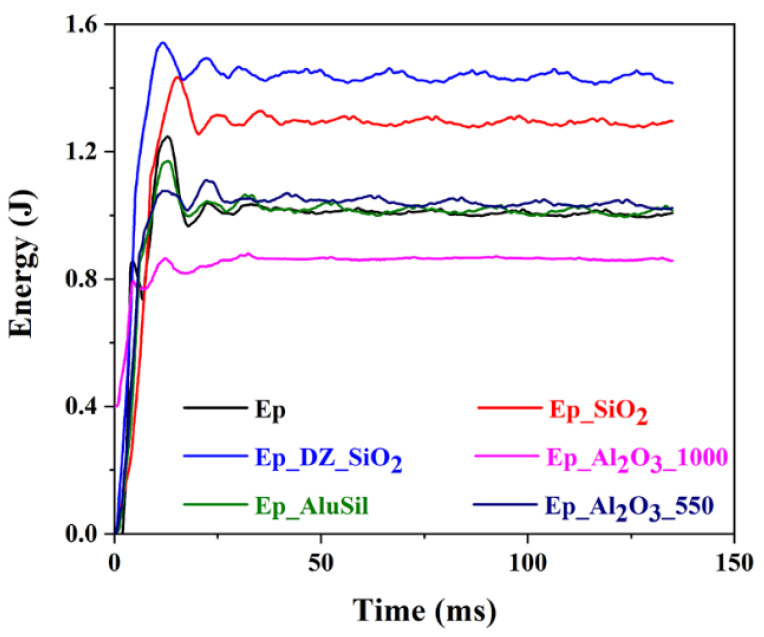
Controlled energy impact testing of epoxy-based composites with different reinforcements.

Controlled energy impact testing enables quantification of the energy absorbed by a material within a defined time interval, which can be interpreted as a measure of toughness. Figure 10 compares the absorbed energy of epoxy-based composites with different reinforcements. The neat epoxy matrix exhibits low energy absorption, consistent with its brittle character. Incorporation of silica-based fillers increases the absorbed energy, suggesting that effective particle-matrix interactions enhance resistance to crack propagation. In contrast, γ-alumina and AluSil reinforcements display behavior like the neat epoxy, indicating limited contribution under rapid loading. Notably, α-alumina particles prepared at elevated temperatures reduce the absorbed energy, likely due to diminished surface activity and weakened interfacial bonding. The mechanical properties of epoxy reinforced with α-Al_2_O_3_ are said to depend mainly on particle load and do not have an influence on the type of fracture of the composite [28]. These results highlight the importance of tailoring particle surface properties to promote matrix interaction and load transfer during impact.

## 3. Conclusions

The findings of this study suggest that the mechanical behavior of DGEBA epoxy composites is strongly influenced by the type of ceramic reinforcement and by particle morphology and crystal structure determined by thermal treatment. Among alumina fillers, γ-Al_2_O_3_ (550 °C) provided the most favorable tensile response, likely due to its high surface area and reactive hydroxyl groups that promote interfacial bonding. In contrast, α-Al_2_O_3_ (1000 °C) increased stiffness but appeared to encourage brittle fracture, suggesting that excessive crystallinity and particle sintering may reduce reinforcement efficiency. Bio-derived silica from rice husk showed better dispersion and adhesion than diatomaceous earth, as reflected in improved toughness and flexural strength.

The hybrid AluSil filler (Al_2_O_3_ + bio-SiO_2_, 3:2 ratio, calcined at 1000 °C) achieved balanced reinforcement, combining the rigidity of alumina with the crack-deflecting capacity of silica. This synergy appeared to enhance toughness, flexural strength, and tensile reliability by reducing agglomeration and improving interfacial adhesion. Overall, these results highlight that the ability of particles to interact effectively with the epoxy matrix—through surface chemistry, morphology, and dispersion—is the most critical factor in achieving improved mechanical performance. Tailoring filler composition and thermal treatment, therefore, remains essential for optimizing the balance between strength, stiffness, and toughness in advanced polymer composites.

## 4. Materials and Methods

The epoxy resin used was diglycidyl ether of bisphenol A (DGEBA; Epoxidharz PRO (A)) with a medium molecular weight ≤ 700 cured with an amine-based hardener (Epoxidharz Härter PRO (B)), both supplied by Bepox d.o.o. Belgrade (Epodex GmbH, Krefeld, Germany). Reinforcement fillers included: Al_2_O_3__550: alumina synthesized via sol–gel process and calcined at 550 °C, Al_2_O_3__1000: alumina synthesized via sol–gel and calcined at 1000 °C, bio SiO_2_: silica derived from rice husk calcined at 800 °C, DZ-SiO_2_: silica obtained from diatomaceous earth, and AluSil: hybrid alumina–silica mixture (Al_2_O_3_:SiO_2_ = 3:2), prepared by sol–gel and calcined at 1000 °C.

### 4.1. Preparation of Reinforcement Fillers

Alumina particles were synthesized from aluminum chlorohydrate (ACH. Al_2_(OH)_5_Cl·2H_2_O) dissolved in demineralized water, gelled, dried, ground, and calcined at 550 °C and 1000 °C. The particles were held at specific temperatures for 4 h, with a heating time of 3 h at a heating rate of 5 °C/min. A narrow particle size distribution, spanning the interval of 0.3 to 1.3 µm, was observed for the synthesized alumina particle [29]. Rice-husk-derived silica was obtained by acid treatment (10% H_2_SO_4_, 80 °C, 3 h), followed by rinsing until a neutral pH was reached. The rice husk was incinerated using a Bunsen burner (blue flame, 1100–1500 °C) under atmospheric air conditions, followed by calcination in a furnace at 800 °C for 4 h. A Nano ZS Zetasizer (Malvern Instruments, Malvern, UK) with a 633 nm He–Ne laser was employed to determine the particle size distribution of the silica obtained from rice husks, yielding values between 235 and 730 nm [30]. Diatomaceous earth samples were collected from the Kolubara Basin, Serbia, and characterized by standard silicate rock analysis. The diameter range of diatomaceous earth particles is 0.4–0.9 μm, as presented in the paper [18]. The diatomaceous earth was used as received. The hybrid AluSil particles were prepared by mixing an ACH solution with bio-SiO_2_ to achieve a molar ratio Al_2_O_3_: SiO_2_ = 3:2. followed by gelation, drying, grinding, and calcination at 1000 °C for 3 h. In our previous work [21], the particle size distribution was found to be between 20 and 60 nm. 

### 4.2. Composite Preparation

The polymer matrix used in this study was a two-component epoxy system. The resin (Component A) is based on a bisphenol A-free (BPA-free) epoxy oligomer, ensuring a more environmentally sustainable formulation. The curing agent (Component B) is a cycloaliphatic amine. The technical specifications of the resin system include an equivalent epoxide number (EEN) of 0.53 eq/100 g, a density of 1.1 g/cm^3^, and a relatively low viscosity of 350 mPa·s at 25 °C, which facilitates efficient filler dispersion. The system is characterized by a prolonged pot life and gelling time of 6 h, allowing for extended processing windows. According to the manufacturer’s recommendations, the specimens were cured for 48–72 h at a controlled temperature of 20 °C. During the processing and curing stages, the relative humidity was maintained below 70% to prevent potential moisture interference with the cross-linking process. Epoxy resin and hardener were mixed in a 2:1 mass ratio according to the manufacturer’s recommendations. Ceramic reinforcement fillers were incorporated at 5 wt.% in regard to resin mass. For each formulation, three specimens were prepared, along with the neat epoxy specimens as controls. 5 wt. % fillers were dispersed using 15 min of ultrasound in a water bath. No surface treatment was applied, as the aim of the study was to evaluate the intrinsic properties of the synthesized and natural fillers. Casting was performed in Teflon molds, and specimens were cured under ambient conditions according to the manufacturer’s instructions for seven days. The annotations for every specimen and its short descriptions are given in Table 3.

### 4.3. Characterization Methods

#### 4.3.1. X-Ray Diffraction (XRD)

The crystalline structure of reinforcement particles was examined using X-ray diffraction (XRD) analysis on an Ultima IV Rigaku (Rigaku Corporation, Tokyo, Japan) equipped with a copper tube for X-ray radiation. The Cu Kα generator operated at 30 kV and 20 mA. The reinforcement particles were scanned over a diffraction angle range of 2θ = 10–80° at a scanning speed of 2°/min.

#### 4.3.2. Field Emission Scanning Electron Microscopy (FESEM)

Morphological analysis of the reinforcement particles as well as the fracture surfaces of composite specimens was performed using field emission scanning electron microscopy (FESEM). Tescan Mira3 XMU electronic microscope operated at 20 kV (Oxford Instruments, Abingdon, UK). This technique enabled detailed observation of particle size, shape, dispersion within the epoxy matrix, and fracture mechanisms.

#### 4.3.3. Fourier Transform Infrared Spectroscopy (FTIR)

Interactions between reinforcement particles and the epoxy matrix were investigated using Fourier transform infrared spectroscopy (FTIR) by a Nicolet 6700 spectrometer (Thermo Scientific, Kwai Chung, N.T., Hong Kong). Spectra were recorded in the range of 4000–500 cm^−1^ to identify functional groups and assess chemical bonding between fillers and the polymer matrix.

#### 4.3.4. Mechanical Testing

For each composite formulation, three specimens were tested, and neat epoxy resin specimens were used as controls. The overall length of the specimens for the mechanical testing (flexural or tensile) was 60 mm, while the width and the thickness of the specimens for the three-point bending test were 10 mm and 2.5 mm, respectively. The neck width of the specimens for the tensile test was 4 mm, while their thickness was 3 mm.

The three-point bending test and the tensile test were carried out using an AG-X Plus Universal Testing Machine (Shimadzu, Kyoto, Japan). The flexural test in the form of the three-point bending method was conducted in accordance with the ASTM D 790 standard. The crosshead displacement rate was 1 mm/min. The span length was 40 mm.

The tensile test was performed in accordance with the ASTM D638 standard. The crosshead displacement rate was 1 mm/min.

#### 4.3.5. Numerical Modeling of Filler Distribution

The stochastic MATLAB routine was generated using particle size distributions consistent with SEM observations and a targeted filler volume fraction of approximately 5 wt. %, serving to visualize the proposed reinforcement mechanism.

#### 4.3.6. Surface Morphological and Fractal Characterization

The quantitative analysis of the fracture surface topography was conducted using a custom-developed numerical routine in MATLAB under the Campus-Wide (CW) license (MathWorks, Natick, MA, USA) version R2024b. The methodology was designed to objectively evaluate the influence of various fillers on the surface complexity by determining the arithmetical mean roughness (R_a_) and the fractal dimension (D_f_) [31].

3D Surface Reconstruction and Roughness Analysis

The SEM micrographs were first pre-processed to ensure consistency by converting them to 8-bit grayscale and removing information bars. A 3D intensity-based reconstruction was performed, where the grayscale intensity value of each pixel (0–255) was mapped to a relative height coordinate (Z) [22]. This transformation created a 3D topographical representation of the fracture surface, as shown in the left panels of Figure 7. From this reconstructed landscape, the arithmetical mean roughness (Ra) was calculated using the following expression (1):

(1) $$R_{a}=\frac{1}{n}\sum_{i=1}^{n}\left|Z_{i}-\underline{Z}\right|$$

where Z_i_ represents the height of an individual pixel and $\underline{Z}$ is the mean surface height. Visualizations were rendered using a high-contrast Jet colormap to enhance perception of topographic features and crack-propagation paths [23].

Fractal Dimension Calculation via Box-Counting Method

To quantify the geometric complexity and tortuosity of the fracture surfaces, the fractal dimension (D_f_) was determined using the box-counting algorithm [32]. The processed images were subjected to edge detection using the Canny operator to identify the boundaries of the fracture features. The resulting binary edge maps were then covered with a series of grids consisting of square boxes of decreasing side lengths (s). For each scale, the number of boxes (N(s)) required to cover the identified edges was recorded.

The fractal dimension was extracted as the absolute slope of the linear regression fit on the log-log plot of N(s) vs. 1/s, according to the relation (2) established by Mandelbrot [33]:

(2) $$\;log\;(N\left(s\right))\;=D_{f}log\left(\frac{1}{s}\right)+C\;$$

where C is a constant. High linearity (R^2^ > 0.98) was maintained across all samples. confirming the self-similar nature of the fracture surfaces. The D_f_ value serves as a numerical indicator of the crack-deflection intensity and the tortuosity of the fracture path [34].

#### 4.3.7. Impact Testing

Controlled energy impact testing was conducted using a Hydroshot HITS-P10 high-speed puncture impact tester (Shimadzu, Kyoto, Japan). The apparatus featured a 10 kN load cell and a 12.7 mm hemispherical striker, with the impact velocity maintained at 1 m/s. Absorbed energy values were automatically derived from the recorded load–displacement data via the integrated software.

## Figures and Tables

**Table 1:** The bending test results.

Specimen	Flexural Strength (MPa)	Flexural Modulus (MPa)
Ep	9.91 ± 0.70	285.79 ± 6.670
Ep_AluSil	50.60 ± 3.71	1688.1 ± 278.3
Ep_Al_2_O_3__550	33.87 ± 3.41	1392.6 ± 152.2
Ep_Al_2_O_3__1000	21.96 ± 3.16	966.73 ± 152.5
Ep_SiO_2_	42.58 ± 0.68	1096.3 ± 76.16
Ep_DZ_SiO_2_	33.43 ± 0.69	925.74 ± 20.77

**Table 3:** Annotations and specimen descriptions used in the research.

Specimen	Description
Ep	Neat epoxy resin (DGEBA, with no reinforcement)
Ep_Al_2_O_3__550	Epoxy reinforced with 5 wt.% alumina synthesized by sol–gel calcined at 550 °C
Ep_Al_2_O_3__1000	Epoxy reinforced with 5 wt.% alumina synthesized by sol–gel calcined at 1000 °C
Ep_SiO_2_	Epoxy reinforced with 5 wt.% silica derived from rice husk, calcined at 800 °C
Ep_DZ_SiO_2_	Epoxy reinforced with 5 wt.% silica obtained from diatomaceous earth
Ep_AluSil	Epoxy reinforced with 5 wt.% hybrid alumina–silica mixture (Al_2_O_3_:SiO_2_ = 3:2) calcined at 1000 °C

## Data Availability

The data presented in this study are available on request from the corresponding author or co-authors. The data are not publicly available.

## Abbreviations

The following abbreviations are used in this manuscript:

DGEBA	Diglycidyl ether of bisphenol A
FTIR	Fourier transform infrared spectroscopy
FESEM	Field emission scanning electron microscopy
XRD	X-ray diffraction
ACH	Aluminum chlorohydrate
